# Construction of Electrochemical Aptamer Sensor Based on Pt-Coordinated Titanium-Based Porphyrin MOF for Thrombin Detection

**DOI:** 10.3389/fchem.2021.812983

**Published:** 2022-01-07

**Authors:** Jiazi Jiang, Quan Cai, Minghan Deng

**Affiliations:** ^1^ Department of Emergency, The First Hospital of China Medical University, Shenyang, China; ^2^ Department of Laboratory, The First Hospital of China Medical University, Shenyang, China

**Keywords:** MOF, aptamer, thrombin, porphyrin, surface modifier

## Abstract

In this work, a Pt-coordinated titanium-based porphyrin metal organic framework (Ti-MOF-Pt) was prepared by embedding single-atom Pt through strong interactions between the four pyrrole nitrogen atoms in the rigid backbone of the porphyrin. The synthesized Ti-MOF-Pt was characterized by TEM, XRD, FTIR and BET. Then, the Ti-MOF-Pt has been used for glassy carbon electrode surface modification and consequently used for construction of a thrombin aptamer sensor. The high surface area provides by MOF and excellent electrochemical property provided by Pt enhance the sensing performance. After optimization of amount of aptamer, hybridization time and specific reaction time, the fabricated aptamer sensor exhibited a linear relationship with the logarithm of the thrombin concentration in the range of 4 pM to 0.2 μM. The detection limit can be calculated as 1.3 pM.

## Introduction

Thrombin is a serine protein hydrolase formed by thrombin precursors, which catalyzes the transformation of fibrinogen into fibrin, promotes blood clotting and regulates coagulation. It is of great significance in revealing the mechanism of tumorigenesis and as an early diagnosis and judgment of healing efficacy (Chen et al., 2020; Zhang et al., 2020; Karimi-Maleh et al., 2022). Since the concentration of thrombin in blood is at the nanomolar level, it is of great importance to establish a simple, rapid and highly sensitive method to detect thrombin (Wang et al., 2019; Fan et al., 2020). A nucleic acid aptamer is a synthetic oligonucleotide chain, either a single-stranded DNA or RNA molecule, that is screened *in vitro* by SELEX technology. Compared to traditional antibodies, nucleic acid aptamers have many similar characteristics, such as high stability and specific binding (Yang et al., 2019). Therefore, aptamers have become a major research focus in life sciences and analytical chemistry (Jamei et al., 2021). The analytical methods used for aptamer sensors mainly include surface plasma resonance (Chang, 2021), quartz crystals microbalance (Goda and Miyahara, 2020), colorimetric methods (Tao et al., 2020), spectroscopic techniques (Sengupta et al., 2020) and electrochemical methods (Yang et al., 2019). Among them, electroanalytical sensor based on aptamer is widely used because of its high selectivity and high sensitivity. However, the construction of electrochemical aptamer sensors directly using commercial electrodes still has some limitations. The performance of the constructed sensors cannot meet the detection requirements. Therefore, modification of commercial electrodes with superior materials followed by aptamer immobilization becomes another option.

Among the many electrode modification materials, MOFs have received a lot of attention as a new type of nanomaterials, which are crystalline materials assembled with metal centers and organic ligands (Duan et al., 2019). Due to the large number of combinations between metal centers and organic ligands, many new MOFs can be synthesized by adjusting metal centers, organic ligands and reaction conditions (Cao et al., 2019; Karimi-Maleh et al., 2021; Wang et al., 2021). These advantages have led to the application of MOFs nanomaterials in gas separation, energy storage and electrochemical analysis. Especially in electrochemical sensing (Dang et al., 2020; Xue et al., 2020), the porous structure of MOFs facilitates the penetration of electrolyte and can promote the rapid reaction between substrate and electrode (Hatamluyi et al., 2020). The adjustable pore size can facilitate the separation of analytes and thus improve the selectivity of the constructed sensors (Zhou et al., 2019, 2020; Fu et al., 2021). The porous structure of MOFs leads to their large surface area, which can expose more active sites and help improve the sensitivity of detection (Huang et al., 2020). The above advantages make MOFs have excellent electrochemical properties, especially in electrochemical sensing, and they are very promising electrode materials for design of electrochemical sensor. Porphyrins are molecules widely found in green plants and are among the most important organic photocatalysts. Porphyrins can be used to develop multi-dentate chelating and bridging ligands by introduction of various ligand groups or by adding different central metals to the porphyrin core (Huang et al., 2019; Liu et al., 2021). Different coordination elements and recently developed synthetic techniques have enabled us to develop porphyrin-based MOFs with different structures (Ma X. et al., 2019). In this work, we anchored single-atom Pt embedded in Pt^II^ tetrakis(4-carboxyphenyl)porphyrin (PtTCPP) through strong interactions between the four pyrrole nitrogen atoms in the rigid backbone of the porphyrin. Ti-oxo clusters were also used as metal nodes to facilitate charge transfer. The results show that the introduction of monatomic Pt into the center of the planar porphyrin skeleton can improve the electrochemical properties of the MOF, which can exhibit excellent sensitivity after the immobilization of aptamer.

## Materials and Methods

### Materials

Methyl 4-formylbenzoate, pyrrole, ethyl acetate, methylene blue (MB), PtCl_2_, benzonitrile potassium ferricyanide, dipotassium hydrogen phosphate, potassium dihydrogen phosphate were purchased from Shanghai Maclean Biochemical Technology Co. Propionic acid, tetrahydrofuran and CH_2_Cl_2_ were purchased from Chengdu Kelong Chemical Reagent Co. Thrombin, bovine serum protein (BSA), and human serum protein (HSA) were purchased from Shanghai Yisheng Biotechnology Co. The oligonucleotide chains were purchased from Suzhou Hongxun Biotechnology Co.

Aptamer: 5′- CACTGTGGTTGGTGT GGTTGG-3′

Capture probe: 5′- CCAACCACAGTG-3′

### Preparation of platinum(II) tetrakis(4-Carboxyphenyl)porphyrin Complex

Methyl 4-formylbenzoate (14.410 g, 0.086 mol) was completely dissolved in propionic acid (250 ml) in a round bottom flask, and then pyrrole (6.09 ml, 0.086 mol) dissolved in 20 ml of propionic acid was slowly added. The mixture was refluxed at 150°C for 12 h. After cooling to room temperature, the obtained precipitates were then treated sequentially with a large amount of ethanol, ethyl acetate and a small amount of tetrakis. The precipitate was washed with a large amount of ethanol, ethyl acetate and a small amount of THF. The collected purple precipitate was dried at 70°C for 12 h and denoted as TMCPP. PtCl_2_(226 mg, 0.854 mM) and TMCPP(362 mg, 0.427 mM) were completely dissolved in 50 ml of anhydrous benzonitrile and refluxed for 36 h under N_2_ gas. After removal of the solvent, the resulting solid was purified by column chromatography using CH_2_Cl_2_/hexane as eluent. The resulting solid was then recrystallized with CH_2_Cl_2_/hexane to obtain PtTMCPP. The obtained PtTMCPP (300 mg, 0.354 mM) was dissolved in a 26 ml (THF/MeOH = 1:1) mixture. 13 ml of KOH (2 M) was then added above solution and kept at reflux for 12 h at 90°C. After cooling to room temperature, some of the solvent was removed and the solution was filtered to obtain a clarified solution, which was then acidified with 1 M HCl until no precipitate formed (pH ≈ 2). The precipitate was collected by centrifugation, washed several times with water, and dried under vacuum at 80°C for 8 h to obtain PtTCPP.

### Preparation of Ti-MOF-Pt

A mixture of 105 μL of tetrabutyl titanate Ti(OBu)_4_, 50 mg of PtTCPP and 2.7 g of benzoic acid was dissolved in 8 ml of N,N-diethylformamide (DEF) at room temperature with stirring. The mixture was transferred to a reaction vessel and heated at 150°C for 5 days. The product was collected by centrifugation and washed several times with acetone to obtain an orange solid Ti-MOFs-Pt. Ti-MOFs were prepared using a similar method except using 5,10,15,20-tetracarboxyphenylporphyrin (H_2_TCPP) to replace the PtTCPP.

### Preparation of Aptamer Sensors

The bare GCE was polished with 0.3 and 0.5 μm Al_2_O_3_ powder in turn, and then cleaned with anhydrous ethanol and distilled water with 5 min sonication. A certain amount of 0.2 mg/ml of Ti-MOF-Pt or Ti-MOFs ethanol dispersion was drop coated on the GCE surface and dried naturally. 40 μL of capture probe (20 μM) was added dropwise to the electrode surface, left overnight and then the electrode was rinsed with PBS buffer (denoted as ss/Ti-MOFs-Pt/GCE). 10 μL of 20 μM aptamer solution was added dropwise to the surface of the modified electrode and left for 1 h at room temperature to allow sufficient hybridization between the aptamer and the probe (ds/Ti-MOFs-Pt/GCE). After rinsing with PBS, the modified electrode was inserted in PBS containing 20 μM MB. MB was modified to the electrode surface by specific adsorption, and finally the electrode was inserted in pH 7.0 buffer solution and deionized water for 10 min each in turn to obtain the aptamer sensor.

### Characterizations

All electrochemical measurements were carried out using a CHI660 electrochemical workstation. A three-electrode system was applied, where a modified GCE, a Pt wire and a saturated silver chloride electrode were used as working electrode, counter electrode and reference electrode, respectively. The XRD analysis was carried out by a XRD-6100 X-ray diffractometer from Shimadzu, Japan. FTIR analysis was carried out by a IR Prestige-21 Infrared Spectrometer from Shimadzu, Japan. BET surface areas and pore size distributions were measured by nitrogen adsorption/desorption on an ASAP 2020 HD88(Micromeritics, GA, United States) adsorption analyzer. Pore size distribution curves were calculated by the Barrett-Joyner-Halenda (BJH) method. TEM was carried out by a JEOL JEM 2100plus. XPS was performed by using the Thermo Escalab spectrometer for characterization.

## Results and Discussion

**FIGURE 1:**
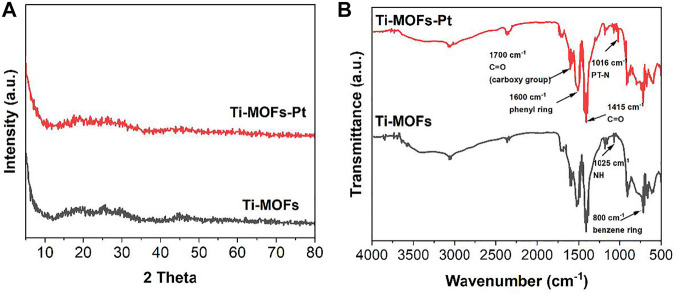
**(A)** XRD patterns and **(B)** FTIR spectra of Ti-MOFs and Ti-MOFs-Pt.

Figure 1A shows the XRD patterns of Ti-MOFs and Ti-MOFs-Pt. It can be seen that both materials show a strong diffraction peak at 2θ = 4.00° with a series broad diffraction peaks at 2θ = 18.50°, 29.80°, and 44.90°, which can be attributed to the successful synthesis of Ti-MOFs. The result is in agreement with the previous report (Feng et al., 2020). Moreover, no characteristic diffraction peaks belonging to Pt nanoparticles were observed in Ti-MOFs-Pt. This does not indicate the absence of Pt formation, probably because of the absence of significant Pt aggregation in Ti-MOFs-Pt. Therefore, the formation of Pt needs to be confirmed by other characterization methods.

The successful synthesis of Pt can be characterized by FTIR. Figure 1B shows the FTIR spectra of Ti-MOFs and Ti-MOFs-Pt. It can be seen that both MOFs have an absorption peak at 1700–700 cm^−1^ that can be attributed to the characteristic absorption band of the porphyrin macrocyclic skeleton (Sadeghi et al., 2016). Usually, the peak near 1,415 cm^−1^ is the stretching vibration of the C=N bond in the pyrrole ring and the peak near 1,600 cm^−1^ is the vibration of the phenyl ring outside the pyrrole ring (Ma et al., 2020). The peak near 800 cm^−1^ is attributed to the characteristic peak of the para-substituted benzene ring (Ma J. et al., 2019), while the peak near 1700 cm^−1^ can be ascribed to the C=O stretching vibration of the carboxyl group (Sadeghi et al., 2016). Notably, a significant decline of the NH peak (at 1,025 cm^−1^) can be observed from Ti-MOFs-Pt compared to Ti-MOFs, along with the appearance of a new Pt-N peak near 1,016 cm^−1^ (Sadeghi et al., 2016), indicating that a single Pt atom has been successfully implanted in the center of the porphyrin molecule.

**FIGURE 2:**
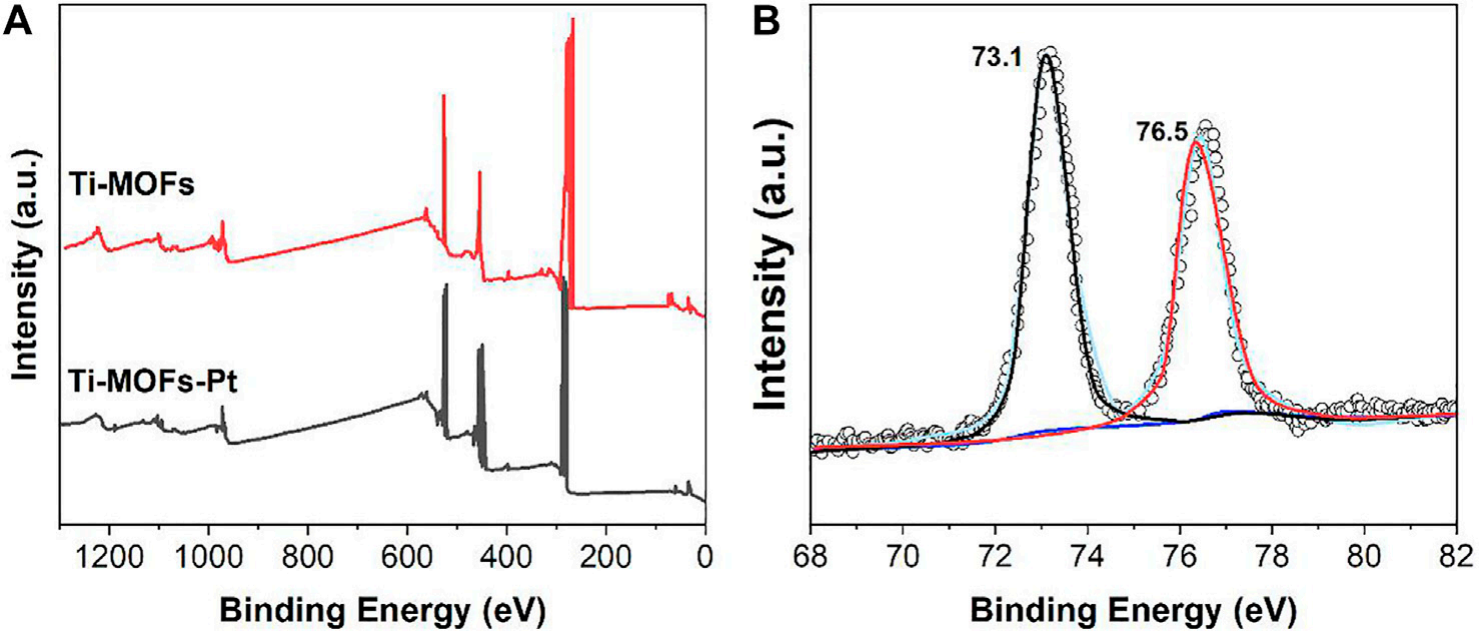
**(A)** XPS full spectra of Ti-MOFs and Ti-MOFs-Pt. **(B)** Pt 4f spectrum of Ti-MOFs-Pt.

The presence of Pt can also be confirmed by XPS characterization. As shown in the XPS spectra (Figure 2A), the pure Pt peak in Ti-MOFs-Pt appears at 74.1 eV, which confirms that Pt atoms have been successfully introduced into Ti-MOFs. The Pt 4f spectrum (Figure 2B) shows two peaks at binding energies of 76.5 and 73.1 eV, corresponding to the 4f_5/2_ and 4f_7/2_, respectively. The peak positions between Pt(II) and Pt(0) suggest that the positively charged Pt atoms can be effectively electron-transferred through enhanced interactions between the porphyrin-linked ligand and the embedded Pt atoms (Chen et al., 2018).

**FIGURE 3:**
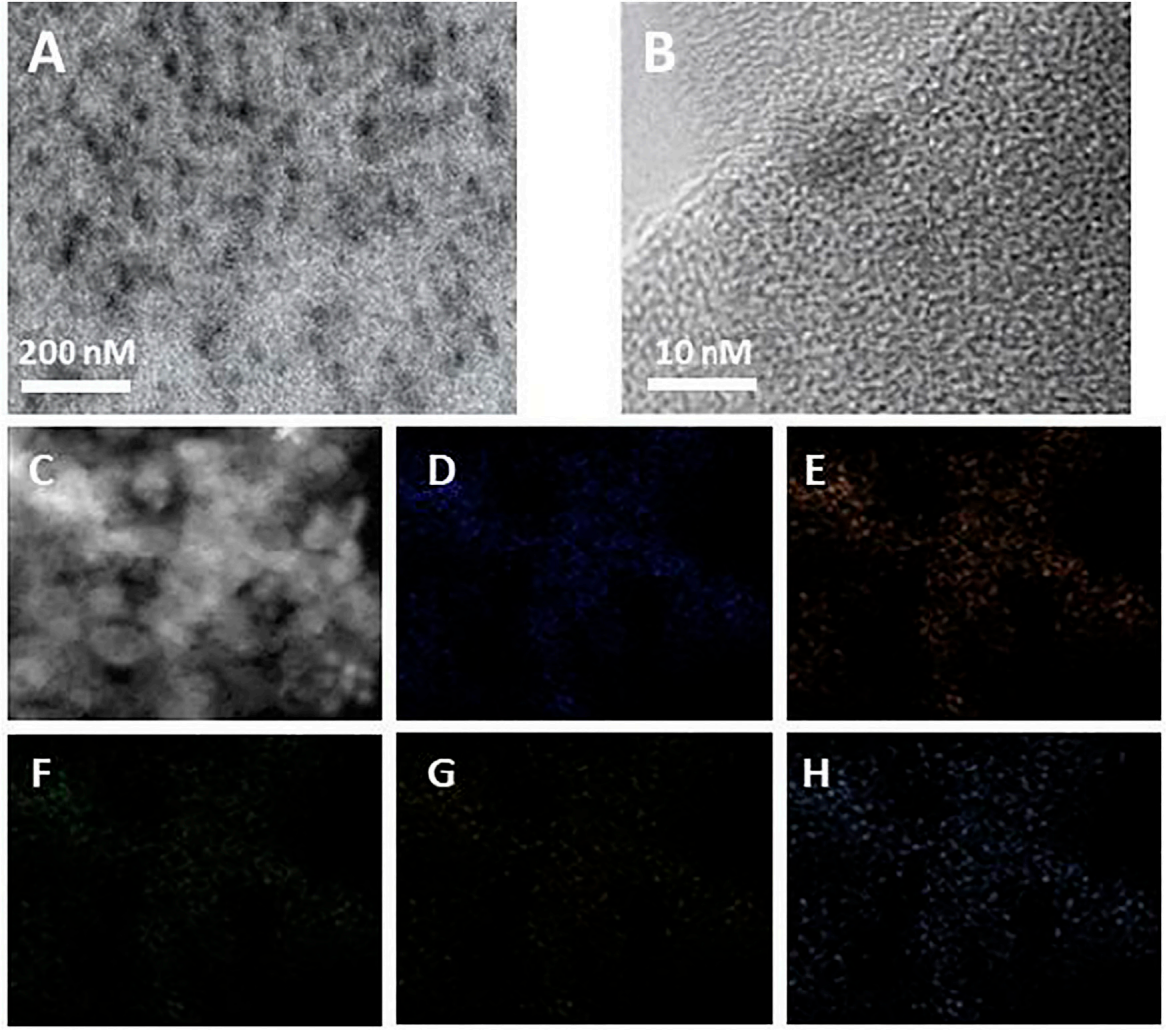
**(A)** TEM and **(B)** HRTEM images of Ti-MOFs-Pt. **(C–H)** C, N, O, Ti, and Pt mapping of Ti-MOFs-Pt.

The morphology of Ti-MOFs-Pt was characterized by TEM and shown in Figure 3A. Ti-MOFs-Pt exhibits a multivacancy morphology with abundant pores to provide more efficient contact interfaces and more exposed surface active sites (Banis et al., 2013). No periodic lattice patterns were observed in the HRTEM image (Figure 3B), suggesting that the individual atoms in Ti-MOFs-Pt have no aggregation. The elemental mapping (Figures 3C–H) shows a homogeneous distribution of C, N, O, Ti, and Pt throughout the selected region, which demonstrates the homogeneity of the various components in the Ti-MOFs-Pt material.

**FIGURE 4:**
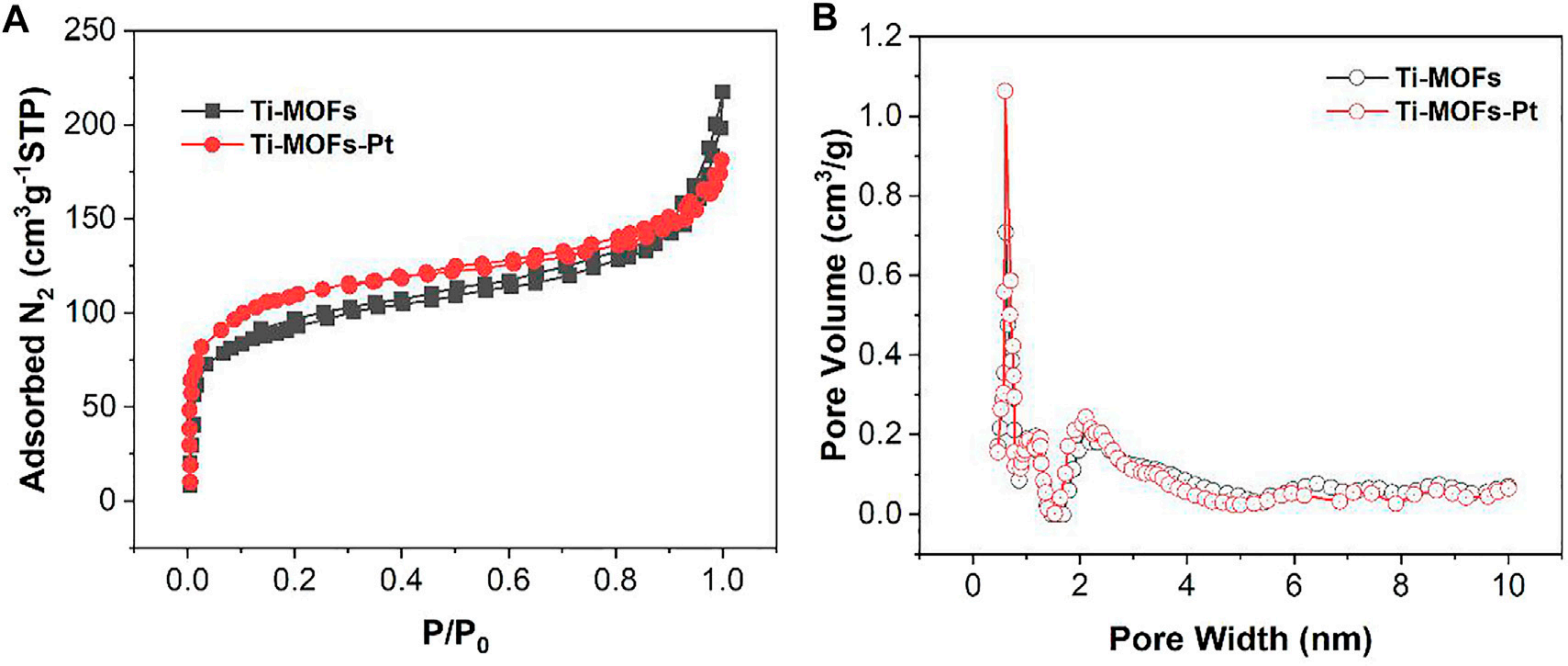
**(A)** N_2_ adsorption-desorption isotherms and **(B)** pore size distribution of Ti-MOFs and Ti-MOFs-Pt.

The properties of Ti-MOFs-Pt pore size were characterized by BET. The nitrogen adsorption-desorption isotherms and the calculated pore size distributions of Ti-MOFs and Ti-MOFs-Pt were shown in Figures 4A,B, respectively. The results demonstrate that the main pores formed in Ti-MOFs and Ti-MOFs-Pt were micropores (<2 nm) and mesopores (2–50 nm). The BET surface areas of Ti-MOFs and Ti-MOFs-Pt were calculated to be 304.2 m^2^/g and 399.7 m^2^/g, respectively, indicating that the introduction of Pt atoms led to the formation of more microchannels in Ti-MOFs, which resulted in the increase of specific surface area (Xu C. et al., 2020).

**FIGURE 5:**
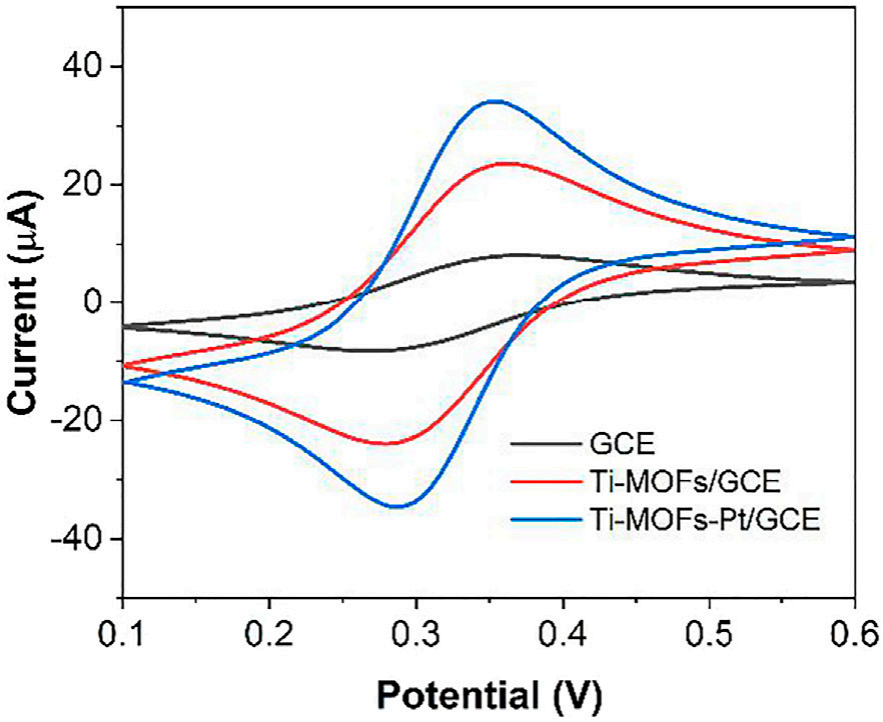
CV of GCE, Ti-MOFs/GCE and Ti-MOFs-Pt/GCE in 0.1 M KCl solution containing 2 mM K_4_Fe(CN)_6_/K_3_Fe(CN)_6_ (scan rate: 50 mV/s).

Figure 5 shows the cyclic voltammograms (CVs) of the three electrodes of GCE, Ti-MOFs/GCE and Ti-MOFs-Pt/GCE in 0.1 M KCl solution containing 2 mM K_4_Fe(CN)_6_/K_3_Fe(CN)_6_. As seen from the figure, the GCE after modification of Ti-MOFs has a slightly larger redox potential difference than GCE, representing that the electron transfer rate on the electrode surface received a little hindrance (Xu Y. et al., 2020). However, the redox current of Ti-MOFs/GCE is much larger than that of GCE, representing that the modification of Ti-MOFs provides additional specific surface area to the electrode that more probe molecules were involved in the electrochemical reaction (Qiu et al., 2019). Similarly, Ti-MOFs-Pt/GCE also exhibited a very significant current, representing that Ti-MOFs-Pt also provided additional specific surface area to the electrode. Further, the redox potential difference of Ti-MOFs-Pt/GCE is smaller than that of GCE, representing that the embedding of Pt increases the conductivity of Ti-MOFs. An excellent electrochemical properties can be expected for Ti-MOFs-Pt/GCE.

**FIGURE 6:**
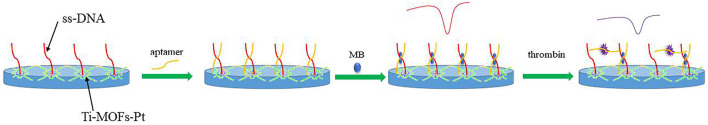
Scheme representation of electrochemical aptamer sensor assembly and detection.

**FIGURE 7:**
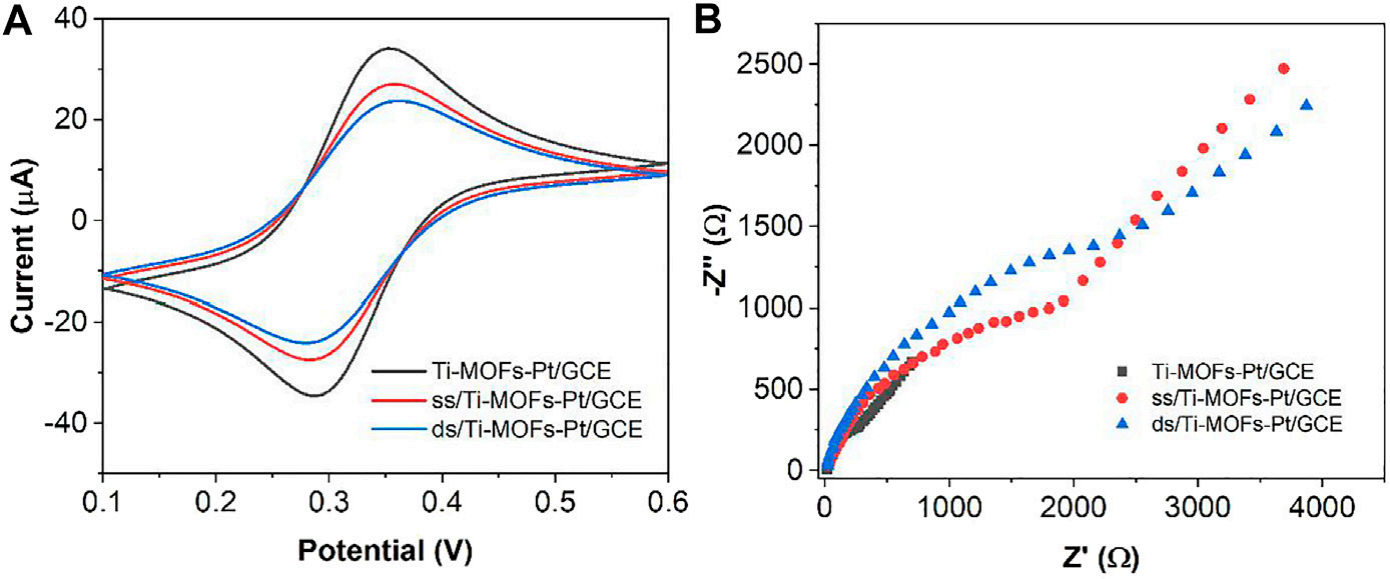
**(A)** CVs and **(B)** EIS of Ti-MOFs-Pt/GCE, ss/Ti-MOFs-Pt/GCE and ds/Ti-MOFs-Pt/GCE in 0.1 M KCl solution containing 2 mM K_4_Fe(CN)_6_/K_3_Fe(CN)_6_.

Figure 6 shows the scheme of the proposed electrochemical aptamer sensor. In the presence of thrombin, the appropriate conformation of thrombin on the surface of the aptamer sensor changes. It can result in the deconvolution of the DNA capture manipulator and the thrombin double-stranded structure on the surface of the sensor, and the release of MB from the double-stranded structure. Figure 7A shows the CV plots of Ti-MOFs-Pt/GCE before and after the modification of the capture probe and thrombin aptamer. It can be seen from the figure that when the capture probe is modified to the electrode surface, the peak current decreases because the negatively charged DNA strand has an electrostatic repulsion with the equally negatively charged [Fe(CN)_6_]^3−/4−^ (Ahour and Ahsani, 2016). When the thrombin aptamer binds to the electrode surface by hybridization reaction with the capture probe, the electrostatic repulsion was enhanced and the peak current decreases due to the further influence of the negatively charged aptamer (Tolouei et al., 2020). These behaviors indicated that both the capture probe and the aptamer are successfully modified to the electrode surface.

Figure 7B shows the Nyquist plots of Ti-MOFs-Pt/GCE, ss/Ti-MOFs-Pt/GCE and ds/Ti-MOFs-Pt/GCE. It can be seen that Ti-MOFs-Pt/GCE has the smallest impedance value. The impedance value gradually increases with the assembly of the capture probe and thrombin aptamer on the electrode surface (Balasubramani et al., 2020; Li et al., 2021). This result agrees with the results of CV described above.

**FIGURE 8:**
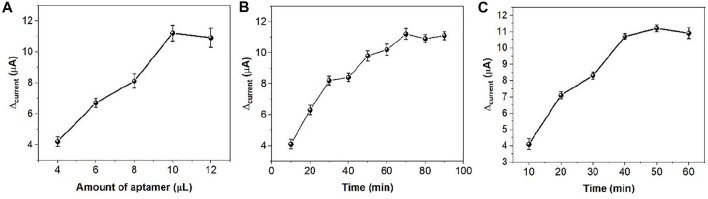
The effect of **(A)** amount of aptamer, **(B)** hybridization time and **(C)** specific reaction time in thrombin sensing.

When ds/Ti-MOFs-Pt/GCE is immersed in MB solution, MB molecules are embedded in the DNA double strand. In the presence of thrombin, the conformation of the aptamer sensor surface changes. The sensor surface captures the probe and the thrombin double-stranded structure will deconvolute, resulting in the release of MB from the double-stranded structure (Taghdisi et al., 2019; Yu et al., 2019). Therefore, the use of electrochemical detection of the change in peak current can be used to detect the concentration of thrombin in solution. The detection sensitivity of the aptamer sensor is influenced by many factors, such as the amount of thrombin aptamer modification, the hybridization time of the capture probe to the aptamer and the specific reaction time of thrombin to the aptamer. We optimized these parameters by using 100 nM thrombin as an analyte. As can be seen from Figure 8A, the measured electrical signal values gradually increase when increasing the amount of aptamer modification, which is due to more aptamers hybridizing with the capture probe to form DNA duplexes, resulting in more MB molecules embedded in the DNA duplex structure. After the amount of aptamer modification exceeded 10 μL, the response values plateaued, which may be due to the saturation of the thrombin aptamer hybridized with the capture probe. Figure 8B demonstrates the effect of the hybridization time of the capture probe with the thrombin aptamer on the signal. The current difference caused by thrombin increases with time from 0 to 60 min. After 60 min, the current difference value remains almost constant. This indicates that the capture probe and the thrombin aptamer react essentially completely within 60 min. Therefore, we chose 60 min as the hybridization time. Figure 8C demonstrates the effect of the time of reaction between thrombin and aptamer on the assay. It can be seen that the current difference increases with time from 0 to 40 min. After 40 min it stabilizes. Therefore, we chose 40 min as the optimal reaction time between thrombin and aptamer.

**FIGURE 9:**
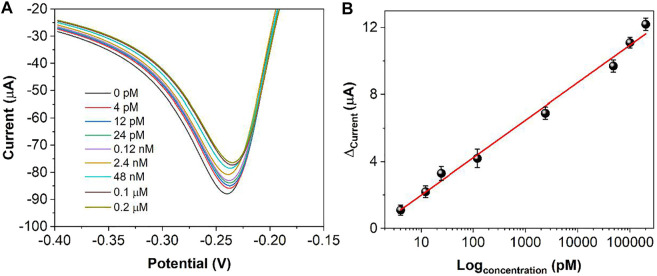
**(A)** DPV curves of ds/Ti-MOFs-Pt/GCE towards different concentrations of thrombin (a to h: 4, 12, 24 pM, 0.12, 2.4, 48 nM, 0.1 μM, 0.2 μM). **(B)** Plots of thrombin against the peak current difference.

Figure 9 demonstrates the DPV curves at different thrombin concentrations. It can be seen that the peak current decreases continuously as the concentration of thrombin increases. In the range of 4 pM to 0.2 μM, the current difference shows a linear relationship with the logarithm of the thrombin concentration. The linear equation is Ip (μA) = 2.2378logc (pM) −0.2374. The detection limit can be calculated as 1.3 pM based on signal to noise of 3 (S/N = 3). Table 1shows the sensing performance of the proposed electrochemical sensor with previous reported works. It can be seen that the reported sensor has competitive performance. In order to test the practical applicability of the ds/Ti-MOFs-Pt/GCE, serum sample have been used as real sample with a standard addition method. The recovery of thrombin was in the range of 95.6–104.1%, with a RSD of less than 6.2%, based on three parallel measurements. The results indicated that the ds/Ti-MOFs-Pt/GCE can satisfy the detection of thrombin in an actual biological environment.

**TABLE 1:** Comparison of different methods for thrombin detection.

Signal marker	Modifier	Linear range	LOD (pM)	Reference
H_2_O_2_	AgNWs + Au-CeO_2_	0.5 pM–30 nM	0.25	Zhang et al. (2020)
MB	Three-stranded DNA complexes	5 pM–1 nM	1.7	Bao et al. (2015)
Bisferrocene	N/A	1.2 pM–12 nM	0.8	Fan et al. (2020)
MB	DNA motifs	50 pM–100 nM	23.6	Yang et al. (2018)
MB	Ti-MOFs-Pt	4 pM–0.2 μM	1.3	This work

**FIGURE 10:**
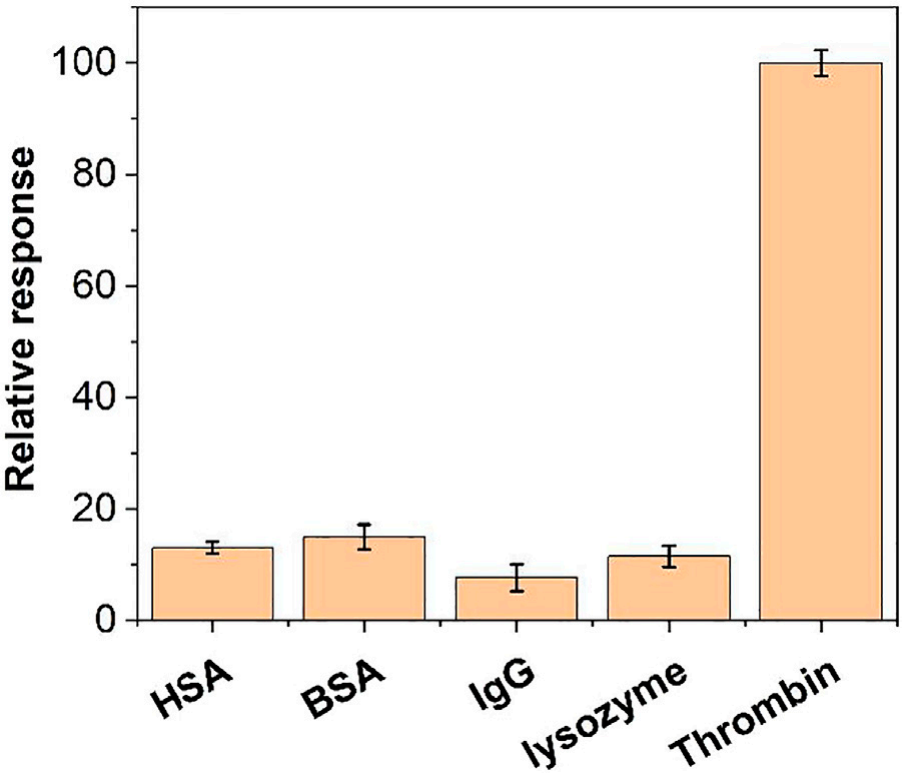
The relative response of the aptamer sensor towards 10 nM thrombin, BSA, IgG, lysozyme and HSA.

We further examined the specificity of this aptamer sensor. We performed tests with BSA, IgG, lysozyme and HSA. As shown in Figure 10, the same concentration of BSA and HSA could not produce a significant DPV electrochemical signal under the same conditions. Therefore, the aptamer sensor has a good selectivity for thrombin.

To examine the reproducibility of the aptamer sensor, we performed six parallel tests with DPV on 10 nM of thrombin. The relative deviation of the measured response currents was 0.62%, indicating that the aptamer sensor has excellent reproducibility. We also tested six individual aptamer sensors and measured a relative deviation of 2.48%, indicating that the sensors are also very reproducible.

## Conclusion

The assembly of the aptamer sensor was characterized and optimized by CV and EIS. The electrocatalytic kinetics of MB on the sensor surface was explored. Under the optimum conditions, a linear relationship with the logarithm of the thrombin concentration can be obtained in the range of 4 pM to 0.2 μM. The detection limit can be calculated as 1.3 pM. The modification of Ti-MOFs-Pt increases the electrocatalytic active area of the aptamer sensor, which facilitates the amplification of the electrical signal and enables the aptamer sensor to have a wide linear detection range and a low detection limit. This methodology also can be extended for the detection of other proteins. In addition, the proposed aptamer sensor has good reproducibility and specificity.

## Data Availability

The original contributions presented in the study are included in the article/Supplementary Material, further inquiries can be directed to the corresponding author.
